# Supplementary material for: Meta-16S rRNA Gene Phylogenetic Reconstruction Reveals the Astonishing Diversity of Cosmopolitan Myxobacteria
Source: Microorganisms. 2019 Nov 11;7(11):551. doi: 10.3390/microorganisms7110551 (PMC6920832; doi:10.3390/microorganisms7110551)
Supplement: Supplementary file 1 [file microorganisms-07-00551-s001.zip › Supplementary Material.pdf]

microorganisms

Supplementary material

The content

Figure S1. The phylogenetic tree inferred from 16S rRNA gene sequences showing the positions of bacteria within the order *Myxococcales*........ 3

Figure S2. The expanded phylogeny of myxobacteria. The sequence of *Desulfovibrio desulfuricans* ATCC 27774 roots the tree.. 5

Figure S3. The percentage of the cultured, the uncultured, and the unknown in different environments........ 6

Table S1. The detailed information of all sequences used in this study........ 7

Table S2. The Hex values and rgb/rgba values of colors used to mark the elements........ 8

Table S3. Sequences numbers and proportion of ten environmental categories. 9

Table S4. Sequence numbers from different countries, areas and the unknown sources. 10

Table S5. The sequence numbers and percentages of 58 families, 445 genera, and 998 species. 11

Table S6. The taxonomy of all type strains within the order Myxococcales from the LPSN database and this study, respectively. 12

Table S7. The occurrence frequencies (%) of new taxa of the three known suborders at the three taxonomic levels. 17

Table S8. The coverage values of the suborder-based and environment-based "samples" at 97% identity 18

Table S9. The environment-specific taxa of myxobacteria at the family, genus, and species levels. 19

Data S1. The newick file of phylogenetic tree from the FastTree software based on 16S rRNA gene sequences. 20

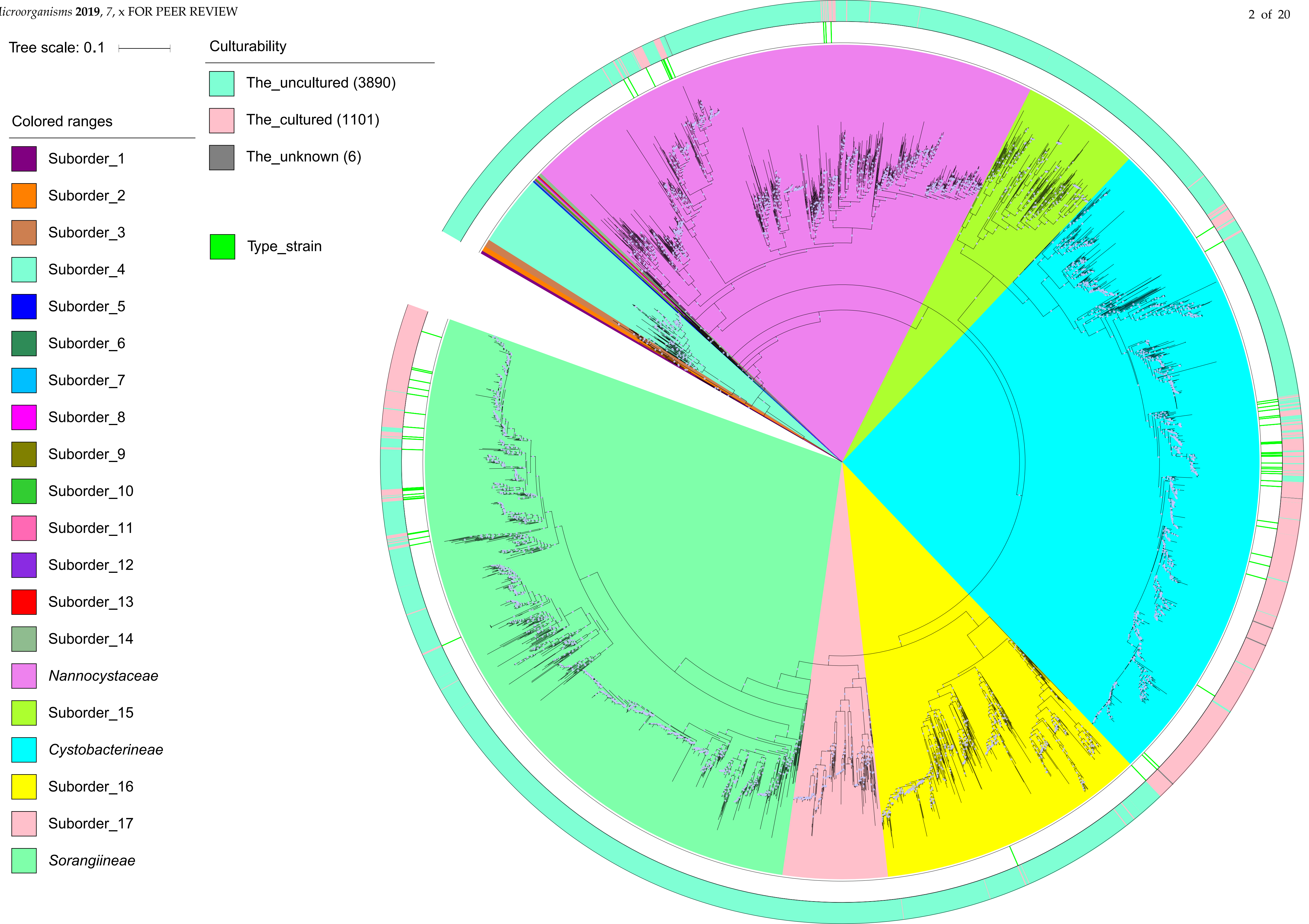
Tree scale: 0.1
Colored ranges
Suborder_1
Suborder_2
Suborder_3
Suborder_4
Suborder_5
Suborder_6
Suborder_7
Suborder_8
Suborder_9
Suborder_10
Suborder_11
Suborder_12
Suborder_13
Suborder_14
Nannocystaceae
Suborder_15
Cystobacterineae
Suborder_16
Suborder_17
Sorangiineae
Culturability
The_uncultured (3890)
The_cultured (1101)
The_unknown (6)
Type_strain

Figure S1. The phylogenetic tree inferred from 16S rRNA gene sequences showing the positions of bacteria within the order *Myxococcales*. The sequence of *Desulfovibrio desulfuricans* ATCC 27774 roots the tree. Scale bar, 0.1 substitutes per nucleotide position. Bootstrap values are indicated by solid circles filled by light blue (rgba(200, 200, 255, 0.8)) with different sizes ranged from 0.5 px to 3 px on the middle (50%) of branches. Suborder-level clusters are indicated by 20 different colors (as shown in Table S1) on the branches of the tree. The culturability of isolates located on the outer ring are indicated by the three different colors (as shown in Table S2). The type strains located on the inner outer are indicated by the color of rgb(0, 255, 0).

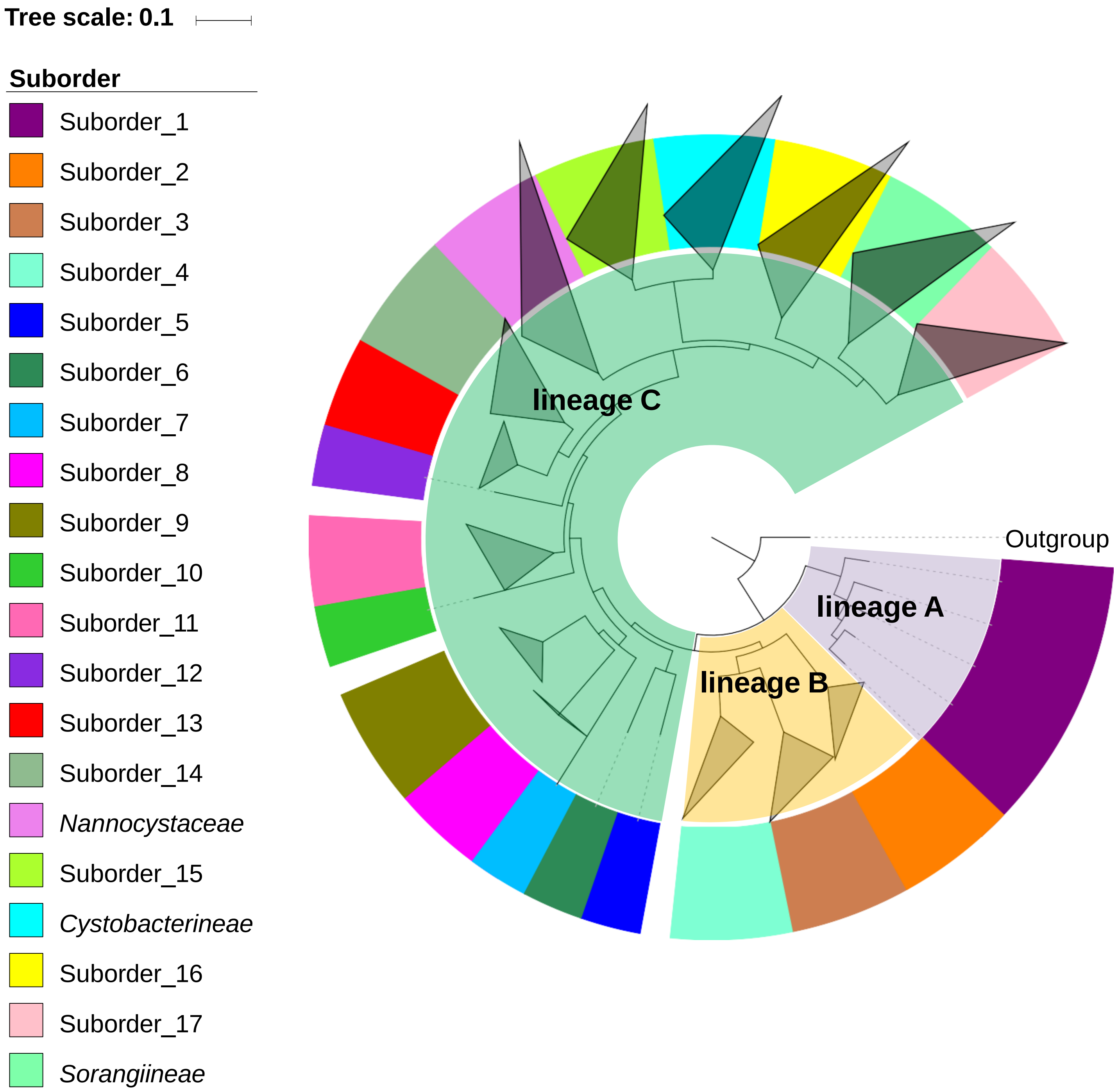
Tree scale: 0.1
Suborder
Suborder_1
Suborder_2
Suborder_3
Suborder_4
Suborder_5
Suborder_6
Suborder_7
Suborder_8
Suborder_9
Suborder_10
Suborder_11
Suborder_12
Suborder_13
Suborder_14
Nannocystaceae
Suborder_15
Cystobacterineae
Suborder_16
Suborder_17
Sorangiineae
lineage C
lineage A
lineage B
Outgroup

Figure S2. The expanded phylogeny of myxobacteria. The sequence of *Desulfovibrio desulfuricans* ATCC 27774 roots the tree. Scale bar, 0.1 substitutions per nucleotide position. Suborder-level clusters are indicated by 20 different colors (as shown in Table S2) on the branches of the tree. Except for the Suborder_1, Suborder_5, Suborder_6, Suborder_7, Suborder_10, and Suborder_12, each of the fourteen remaining suborders is collapsed to a single branch of the tree represented by grey triangles. The lineage A congruent with the Suborder_1 is marked by the color of "#B3A2C799" or rgba(179, 162, 199, 0.6). The lineage B consisted of Suborder_2 to Suborder_4 is marked by the color of "#FFC00099" or rgba(255, 192, 0, 0.6). The lineage C contains the remaining sixteen suborders was marked by the color of "#00B05099" or rgba(0, 176, 80, 0.6).

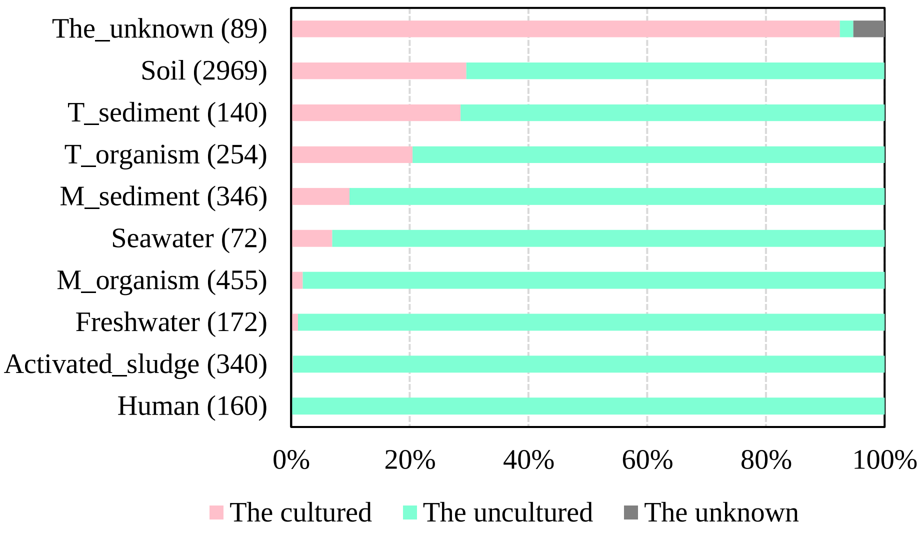

Figure S3. The percentage of the cultured, the uncultured, and the unknown in different environments

Table S1. The detailed information of all sequences used in this study

Table S2. The Hex values and rgb/rgba values of colors used to mark the elements

**Suborder**	**Color**	**Hex color value**	**RGB value**
Suborder_1		#800080	128, 0, 128
Suborder_2		#cd853f	205, 133, 63
Suborder_3		#FF7F50	255, 127, 80
Suborder_4		#7fffd4	127, 255, 212
Suborder_5		#0000ff	0, 0, 255
Suborder_6		#2E8B57	46, 139, 87
Suborder_7		#00BFFF	0, 191, 255
Suborder_8		#FF00FF	255, 0, 255
Suborder_9		#808000	128, 128, 0
Suborder_10		#32CD32	50, 205, 50
Suborder_11		#FF69B4	255, 105, 180
Suborder_12		#8A2BE2	138, 43, 226
Suborder_13		#FF0000	255, 0, 0
Suborder_14		#8FBC8F	143, 188, 143
Nannocystaceae		#ee82ee	238, 130, 238
Suborder_15		#adff2f	173, 255, 47
Cystobacterineae		#00FFFF	0, 255, 255
Suborder_16		#FFFF00	255, 255, 0
Suborder_17		#FFC0CB	255, 192, 203
Sorangiineae		#7FFFAA	127, 255, 170
**Environmental categories**			
Soil		#A0522D	160, 82, 45
T_sediment		#FFA500	255, 165, 0
Activated sludge		#FFD700	255, 215, 0
T_organism		#FFFF00	255, 255, 0
Freshwater		#00FF00	0, 255, 0
Human		#FF00FF	255, 0, 255
M_organism		#00FFFF	0, 255, 255
M_sediment		#00BFFF	0, 191, 255
Seawater		#0000FF	0, 0, 255
The_unknown		#808000	128, 128, 0
**Culturability**			
The_cultured		#FFC0CB	255, 192, 203
The_uncultured		#7fffd4	127, 255, 212
The_unknown		#808080	128, 128, 128
**Lineage**			**RGBA value**
Lineage A		#B3A2C799	179, 162, 199, 0.6
Lineage B		#FFC00099	255, 192, 0, 0.6
Lineage C		#00B05099	0, 176, 80, 0.6

Table S3. Sequences numbers and proportion of ten environmental categories.

Environmental categories	Numbers	Proportion
Soil	2969	59.42%
M_organism	455	9.11%
M_sediment	346	6.92%
Activated_sludge	340	6.80%
T_organism	254	5.08%
Freshwater	172	3.44%
Human	160	3.20%
T_sediment	140	2.80%
The_unknown	89	1.78%
Seawater	72	1.44%

Table S4. Sequence numbers from different countries, areas and the unknown sources.

Countries/Areas	Numbers	Countries/Areas	Numbers
China	947	Iran	6
Denmark	939	Malaysia	6
USA	908	**Mediterranean Sea**	6
Mexico	510	Netherlands	6
Panama	187	Pakistan	6
Japan	168	Austria	5
India	140	Czech Republic	5
Germany	113	**Indian Ocean**	5
Spain	101	Namibia	5
France	100	Norway	5
**The unknown**	84	Oman	5
South Korea	75	Belgium	4
**Pacific Ocean**	74	Brazil	4
Singapore	59	Chile	4
**Antarctica**	32	Kazakhstan	4
Tunisia	26	Sri Lanka	4
Finland	24	Uruguay	4
United Kingdom	29	Kenya	3
Canada	22	Nepal	3
Italy/Portugal	22/22	Philippines	3
**Gulf of Mexico**	19	Saint Lucia, Syria	3
Sweden	19	Vietnam	3
**Arctic**	17	Guatemala	2
South Africa	15	Hungary	2
Greece	14	Israel	2
Costa Rica	13	Kiribati	2
**Atlantic Ocean**	12	Morocco	2
Bahamas	12	Nigeria	2
Indonesia	11	Slovakia	2
Puerto Rico	11	Argentina	1
Tanzania	10	**Black Sea**	1
Australia	9	Liberia	1
Egypt	9	Malawi	1
Switzerland	9	Malta	1
Thailand	9	Niger	1
Poland	8	Romania	1
**Red Sea**	8	Saudi Arabia	1
New Zealand	7	Turkey	1
Russia	7	Ukraine	1
Bulgaria	6	Republic of Congo/Zambia	1/1

Note: The identified sea areas and the unknown sources were shown in bold.

Table S5. The sequence numbers and percentages of 58 families, 445 genera, and 998 species.

Table S6. The taxonomy of all type strains within the order Myxococcales from the LPSN database and this study, respectively.

Type_strain	Accession	Taxonomy (Suborder; family; genus; sepecies)	
		**The LPSN database**	**This study**
2CP-1	CP001359.1	*Cystobacterineae*; *Anaeromyxobacteraceae*; *Anaeromyxobacter*; *Anaeromyxobacter dehalogenans*	*Cystobacterineae*; Family_4; Genus_3; Species_8
Cb M10	HE582768	*Cystobacterineae*; *Archangiaceae*; *Archangium*; *Archangium disciforme*	*Cystobacterineae*; Family_6; Genus_27; Species_3
M18	CP011509.1	*Cystobacterineae*; *Archangiaceae*; *Archangium*; *Archangium gephyra*	*Cystobacterineae*; Family_6; Genus_27; Species_3
Cb m2	DQ768113	*Cystobacterineae*; *Archangiaceae*; *Archangium*; *Archangium minus*	*Cystobacterineae*; Family_6; Genus_27; Species_3
Cb vi61	DQ768114	*Cystobacterineae*; *Archangiaceae*; *Archangium*; *Archangium violaceum*	*Cystobacterineae*; Family_6; Genus_27; Species_3
Cb a1	DQ768107	*Cystobacterineae*; *Archangiaceae*; *Cystobacter*; *Cystobacter* armeniaca	*Cystobacterineae*; Family_6; Genus_27; Species_4
Cb b2	DQ768108	*Cystobacterineae*; *Archangiaceae*; *Cystobacter*; *Cystobacter badius*	*Cystobacterineae*; Family_6; Genus_27; Species_4
Cb fe18	DQ768112	*Cystobacterineae*; *Archangiaceae*; *Cystobacter*; *Cystobacter ferrugineus*	*Cystobacterineae*; Family_6; Genus_27; Species_4
M29	ANAH02000068	*Cystobacterineae*; *Archangiaceae*; *Cystobacter*; *Cystobacter fuscus*	*Cystobacterineae*; Family_6; Genus_27; Species_4
Cb g1	DQ768110	*Cystobacterineae*; *Archangiaceae*; *Cystobacter*; *Cystobacter gracilis*	*Cystobacterineae*; Family_6; Genus_27; Species_2
Cb a24t	DQ768111	*Cystobacterineae*; *Archangiaceae*; *Cystobacter*; *Cystobacter miniatus*	*Cystobacterineae*; Family_6; Genus_27; Species_4
Cb v34	DQ768115	*Cystobacterineae*; *Archangiaceae*; *Cystobacter*; *Cystobacter velatus*	*Cystobacterineae*; Family_6; Genus_27; Species_4
NOCB-2	JMCB01000038	*Cystobacterineae*; *Archangiaceae*; *Hyalangium*; *Hyalangium minutum*	*Cystobacterineae*; Family_6; Genus_27; Species_2

M155	DQ768126	Cystobacterineae; Archangiaceae; *Melittangium*; *Melittangium lichenicola*	*Cystobacterineae*; Family_6; Genus_27; Species_4
Me b7	AJ233907	*Cystobacterineae*; *Archangiaceae*; *Melittangium*; *Melittangium alboraceum*	*Cystobacterineae*; Family_6; Genus_27; Species_4
Me b8	CP022163.1	*Cystobacterineae*; *Archangiaceae*; *Melittangium*; *Melittangium boletus*	*Cystobacterineae*; Family_6; Genus_27; Species_4
M 15	FOAP01000045	*Cystobacterineae*; *Archangiaceae*; *Stigmatella*; *Stigmatella aurantiaca*	*Cystobacterineae*; Family_6; Genus_27; Species_2
M26	FNOH01000052	*Cystobacterineae*; *Archangiaceae*; *Stigmatella*; *Stigmatella erecta*	*Cystobacterineae*; Family_6; Genus_27; Species_5
Sg h20	DQ768129	*Cystobacterineae*; *Archangiaceae*; *Stigmatella*; *Stigmatella hybrida*	*Cystobacterineae*; Family_6; Genus_27; Species_2
MCy10943	KX430042	*Cystobacterineae*; *Archangiaceae*; *Vitiosangium*; *Vitiosangium cumulatum*	*Cystobacterineae*; Family_6; Genus_27; Species_5
MCy10944	KX430041	*Cystobacterineae*; *Archangiaceae*; *Vitiosangium*; *Vitiosangium subalbum*	*Cystobacterineae*; Family_6; Genus_27; Species_5
MCy1366	KF767690	*Cystobacterineae*; *Myxococcaceae*; *Aggregicoccus*; *Aggregicoccus edonensis*	*Cystobacterineae*; Family_6; Genus_25; Species_4
M2	CP003389.1	*Cystobacterineae*; *Myxococcaceae*; *Corallococcus*; *Corallococcus coralloides*	*Cystobacterineae*; Family_6; Genus_27; Species_5
Cc e167	AJ811598	*Cystobacterineae*; *Myxococcaceae*; *Corallococcus*; *Corallococcus exiguus*	*Cystobacterineae*; Family_6; Genus_27; Species_5
Cc m8	CP022203.1	*Cystobacterineae*; *Myxococcaceae*; *Corallococcus*; *Corallococcus macrosporus*	*Cystobacterineae*; Family_6; Genus_27; Species_5
M17	FOIB01000024	*Cystobacterineae*; *Myxococcaceae*; *Myxococcus*; *Myxococcus fulvus*	*Cystobacterineae*; Family_6; Genus_27; Species_5
Mx s8	CP004025.1	*Cystobacterineae*; *Myxococcaceae*; *Myxococcus*; *Myxococcus stipitatus*	*Cystobacterineae*; Family_6; Genus_27; Species_5

M22	DQ768119	*Cystobacterineae; Myxococcaceae; Myxococcus; Myxococcus virescens*	*Cystobacterineae*; Family_6; Genus_27; Species_5
FB	DQ768116	*Cystobacterineae; Myxococcaceae; Myxococcus; Myxococcus xanthus*	*Cystobacterineae*; Family_6; Genus_27; Species_5
An D47t	DQ768123	*Cystobacterineae; Myxococcaceae; Pyxidicoccus; Pyxidicoccus fallax*	*Cystobacterineae*; Family_6; Genus_27; Species_5
MCy10636	MH094235	*Cystobacterineae; Myxococcaceae; Simulacricoccus; Simulacricoccus ruber*	*Cystobacterineae*; Family_6; Genus_26; Species_3
B00001	CP012332.1	*Cystobacterineae; Vulgatibacteraceae; Vulgatibacter; Vulgatibacter incomptus*	*Cystobacterineae*; Family_5; Genus_9; Species_2
SMP-2	CP001804.1	*Nannocystaceae; Haliangiaceae; Haliangium; Haliangium ochraceum*	*Nannocystaceae*; Family_10; Genus_4; Species_1
SMP-10	AB062751	*Nannocystaceae; Haliangiaceae; Haliangium; Haliangium tepidum*	*Nannocystaceae*; Family_10; Genus_4; Species_2
Pl vt1	AJ233944	*Nannocystaceae; Kofleriaceae; Kofleria; Kofleria flava*	*Nannocystaceae*; Family_10; Genus_4; Species_2
SHK-1	AB097590	*Nannocystaceae; Nannocystaceae; Enhygromyxa; Enhygromyxa salina*	*Nannocystaceae*; Family_7; Genus_14; Species_6
Na e1	NETK01000001.1	*Nannocystaceae; Nannocystaceae; Nannocystis; Nannocystis exedens*	*Nannocystaceae*; Family_7; Genus_23; Species_1
MNa11734	KY381122	*Nannocystaceae; Nannocystaceae; Nannocystis; Nannocystis konarekensis*	*Nannocystaceae*; Family_7; Genus_23; Species_1
Na p29	GU207878	*Nannocystaceae; Nannocystaceae;* Nannocystis; *Nannocystis* pusilla	*Nannocystaceae*; Family_7; Genus_23; Species_1
SIR-1	ABCS01000069	*Nannocystaceae; Nannocystaceae; Plesiocystis; Plesiocystis pacifica*	*Nannocystaceae*; Family_7; Genus_14; Species_1
Na a1	AB303310	*Nannocystaceae; Nannocystaceae; Pseudenhygromyxa; Pseudenhygromyxa salsuginis*	*Nannocystaceae*; Family_7; Genus_14; Species_3

B00002	CP012333.1	*Sorangiineae; Labilitrichaceae; Labilithrix; Labilithrix luteola*	*Sorangiineae*; Family_5; Genus_11; Species_1
SBNa008	CP016211.1	*Sorangiineae; Phaselicystidaceae; Minicystis; Minicystis rosea*	*Sorangiineae*; Family_5; Genus_16; Species_3
SBKo001	EU545827	*Sorangiineae; Phaselicystidaceae; Phaselicystis; Phaselicystis flava*	*Sorangiineae*; Family_5; Genus_16; Species_5
SBSr002	GU249609	*Sorangiineae; Polyangiaceae; Aetherobacter; Aetherobacter fasciculatus*	*Sorangiineae*; Family_5; Genus_18; Species_9
SBSr003	GU249610	*Sorangiineae; Polyangiaceae; Aetherobacter; Aetherobacter rufus*	*Sorangiineae*; Family_5; Genus_18; Species_9
By c2	BBEO01000805	*Sorangiineae; Polyangiaceae; Byssovorax; Byssovorax cruenta*	*Sorangiineae*; Family_5; Genus_18; Species_7
Cm a14	AJ233938	*Sorangiineae; Polyangiaceae; Chondromyces; Chondromyces apiculatus*	*Sorangiineae*; Family_5; Genus_18; Species_3
Cm c5	CP012159.1	*Sorangiineae; Polyangiaceae; Chondromyces; Chondromyces crocatus*	*Sorangiineae*; Family_5; Genus_18; Species_3
Sy t2	AJ233939	*Sorangiineae; Polyangiaceae; Chondromyces; Chondromyces lanuginosus*	*Sorangiineae*; Family_5; Genus_18; Species_3
Cm p51	GU207875	*Sorangiineae; Polyangiaceae; Chondromyces; Chondromyces pediculatus*	*Sorangiineae*; Family_5; Genus_18; Species_3
Cm a13	AJ233942	*Sorangiineae; Polyangiaceae; Chondromyces; Chondromyces robustus*	*Sorangiineae*; Family_5; Genus_18; Species_3
Pl t4	GU207876	*Sorangiineae; Polyangiaceae; Jahnella; Jahnella thaxteri*	*Sorangiineae*; Family_5; Genus_18; Species_3
Pl sm5	GU207879	*Sorangiineae; Polyangiaceae; Polyangium; Polyangium fumosum*	*Sorangiineae*; Family_5; Genus_16; Species_7
Pl fu5	GU207880	*Sorangiineae; Polyangiaceae; Polyangium; Polyangium sorediatum*	*Sorangiineae*; Family_5; Genus_16; Species_7
Pl s12	GU207881	*Sorangiineae; Polyangiaceae; Polyangium; Polyangium spumosum*	*Sorangiineae*; Family_5; Genus_16; Species_7
MSr9521	KT591707	*Sorangiineae; Polyangiaceae; Racemicystis; Racemicystis crocea*	*Sorangiineae*; Family_5; Genus_18; Species_2
MSr11462	KX443485	*Sorangiineae; Polyangiaceae; Racemicystis; Racemicystis persica*	*Sorangiineae*; Family_5; Genus_18; Species_2
Soce 176	MG824979	*Sorangiineae; Polyangiaceae; Sorangium; Sorangium ambruticinum*	*Sorangiineae*; Family_5; Genus_19; Species_3

Soce 1078	MG824983	*Sorangiineae*; *Polyangiaceae*; *Sorangium*; *Sorangium arenae*	*Sorangiineae*; Family_5; Genus_19; Species_3
Soce 321	MG824980	*Sorangiineae*; *Polyangiaceae*; *Sorangium*; *Sorangium bulgaricum*	*Sorangiineae*; Family_5; Genus_19; Species_3
Soce 1871	EU240497	*Sorangiineae*; *Polyangiaceae*; *Sorangium*; *Sorangium cellulosum*	*Sorangiineae*; Family_5; Genus_19; Species_3
Soce 362	MG824981	Sorangiineae; *Polyangiaceae*; *Sorangium*; *Sorangium dawidii*	*Sorangiineae*; Family_5; Genus_19; Species_3
Soce 375	MG824982	*Sorangiineae*; *Polyangiaceae*; *Sorangium*; *Sorangium kenyense*	*Sorangiineae*; Family_5; Genus_19; Species_3
Soce GT47	MG824978	*Sorangiineae*; *Polyangiaceae*; *Sorangium*; *Sorangium orientale*	*Sorangiineae*; Family_5; Genus_19; Species_3
Soce 1828	MG824984	*Sorangiineae*; *Polyangiaceae*; *Sorangium*; *Sorangium reichenbachii*	*Sorangiineae*; Family_5; Genus_19; Species_3
NOSO-4	CP011125.1	*Sorangiineae*; *Sandaracinaceae*; *Sandaracinus*; *Sandaracinus amylolyticus*	Suborder_16; Family_4; Genus_8; Species_3

Table S7. The occurrence frequencies (%) of new taxa of the three known suborders at the three taxonomic levels.

**Suborder**	**Family**	**Genus**	**Species**
Nannocystaceae	81.82	98.09	98.06
Cystobacterineae	50.00	90.00	93.89
Sorangiineae	80.00	92.00	95.38

Table S8. The coverage values of the suborder-based and environment-based "samples" at 97% identity

**Suborders**	Coverage	**Environments**	Coverage
Sorangiineae	0.862354	The_unknown	0.865169
Suborder_17	0.729469	T_sediment	0.521429
Suborder_16	0.746717	T_organism	0.468504
Cystobacterineae	0.896084	Soil	0.811721
Suborder_15	0.605932	Seawater	0.486111
Nannocystaceae	0.671456	M_sediment	0.595376
Suborder_4	0.763889	M_organism	0.837363
		Human	0.643750
		Freshwater	0.470930
		Activated_sludge	0.688235

Table S9. The environment-specific taxa of myxobacteria at the family, genus, and species levels.

Data S1. The newick file of phylogenetic tree from the FastTree software based on 16S rRNA gene sequences.

© 2019 by the authors. Submitted for possible open access publication under the terms and conditions of the Creative Commons Attribution (CC BY) license (http://creativecommons.org/licenses/by/4.0/).
